# Enhancing glioblastoma therapy: unveiling synergistic anticancer effects of Onalespib - radiotherapy combination therapy

**DOI:** 10.3389/fonc.2025.1451156

**Published:** 2025-01-30

**Authors:** Julia Uffenorde, Mehran Hariri, Eleftherios Papalanis, Annika Staffas, Josefine Berg, Bo Stenerlöw, Hanna Berglund, Christer Malmberg, Diana Spiegelberg

**Affiliations:** 1 Department of Surgical Sciences, Uppsala University, Uppsala, Sweden; 2 Department of Immunology, Genetics and Pathology, Uppsala University, Uppsala, Sweden; 3 Department of Medical Sciences, Uppsala University, Uppsala, Sweden

**Keywords:** CNS tumors, synergy, heat shock protein, radiotherapy, combination therapy, proteomics, proximity extension assay

## Abstract

**Background:**

Glioblastoma (GBM) is the deadliest form of brain cancer, impacting both adults and children, marked by exceptionally high morbidity and mortality rates, even with current standard treatments such as surgery, radiation therapy, and chemotherapy. Therefore, there is a pressing need for new therapeutic strategies to improve survival and reduce treatment side effects. In this study, we investigated the effect of HSP90 inhibition in combination with radiotherapy in established and patient-derived glioblastoma cell lines.

**Methods:**

Potential radiosensitizing effects of the HSP90 inhibitor Onalespib were studied in XTT and clonogenic survival assays as well as in tumor-mimicking multicellular spheroid models. Further, migration capacity and effects on protein expression were studied after exposure to Onalespib and radiation using Proximity Extension Assay analysis.

**Results:**

HSP90 inhibition with Onalespib synergistically enhanced the radiosensitivity of glioblastoma cells grown in 2D and 3D models, resulting in increased cell death, reduced migration capacity and activation of the apoptotic signaling pathway. The proteomic analysis of glioblastoma cells treated with Onalespib, radiation, and their combination revealed significant alterations in protein expression profiles, involved in growth signaling, immune modulation pathways and angiogenesis. Moreover, the combination treatment indicated potential for enhancing cell cycle arrest and apoptosis, suggesting promising anti-tumor effects.

**Conclusion:**

These findings demonstrate that HSP90 inhibition may be a promising strategy to enhance the efficacy of radiotherapy in the treatment of GBM, potentially leading to improved outcomes for patients battling this challenging disease.

## 1 Introduction

Glioblastoma (GBM) is the most frequent primary brain tumor in adults, with a median survival of less than 15 months despite aggressive treatment (1). Its occurrence in children remains relatively rare, constituting 3–15% of primary central nervous system (CNS) tumors. Despite the relative rarity, pediatric GBM exacts a significant toll with high morbidity and mortality rates, and with a 5-year survival of less than 20% (2, 3). The therapeutic strategies of GBM include open surgery and a combination of radiotherapy (60 Gy), typically given over 6 weeks (in 30 fractions of 2 Gy) with concurrent administration of the oral alkylating agent temozolomide (TMZ) (4, 5). There are indications that patients with epigenetic silencing of the DNA-repair protein MGMT in the tumor tissue benefit the most from TMZ, however, pediatric GBMs seldom display methylated MGMT promoters (5, 6). Unfortunately, TMZ treatment often leads to emergent tumor resistance (7), with multiple studies indicating that inactivation of the mismatch repair function (MMR) may be an important mechanism underlying acquired resistance. TMZ produces O6-methylguanine (O6-MG) lesions, which leads to base mispairing with thymine instead of cytosine during DNA replication, triggering DNA repair, cell cycle arrest, and ultimately cell death. In the case of MMR inactivation in post-treatment GBM patients, O6-MG is not recognized by MMR proteins and bypasses apoptosis, resulting in the survival of cancer cells and the proliferation of “cytidine to thymidine” hypermutator phenotypes (8, 9). Despite these insights, many aspects involved in GBM resistance to treatment are still poorly understood. The inadequate killing of cancer stem cells and the upregulation of DNA damage response (DDR) have been described as important contributors to low cancer survival (10).

New treatment approaches are needed to increase therapy success rates and improve clinical outcomes for patients with GBM. Based on the current understanding of the mechanisms underlying radiotherapy resistance, this may involve specific targeting of the resistant cancer cell subpopulations, as well as DDR mechanisms.

Recent research has identified the molecular chaperone heat shock protein 90 (HSP90) as a promising target for improving radiation treatment, including GBM (11–16). HSP90 is a member of the heat-shock protein family with a molecular mass of 90 kD. HSP90 is often overexpressed in human tumors, having a central role in buffering cellular stress and protein folding in an ATP-dependent manner. For this, HSP90 stabilizes multiple DDR proteins and oncoproteins which helps ensuring tumor cell survival and proliferation (17). HSP90 inhibitors exhibit higher affinity for the intertumoral HSP90 compared to the HSP90 in normal cells. This is due to the increased ATPase activity of HSP90 in tumor cells, which results from mutations or deregulation that are commonly present in cancerous cells (18). Therefore, HSP90 inhibitors have received interest as potentially attractive and potent cancer treatment agents. In our study, we used Onalespib, a second-generation HSP90 inhibitor with favorable toxicity profile (19) and the benefit of penetrating the blood-brain barrier (20). Onalespib already has undergone phase I studies with solid tumors with acceptable toxicity profiles and has shown antitumor activity in combination treatment (19, 21). Furthermore, long-acting effects of Onalespib against gliomas with a decrease in proliferation, migration, and angiogenesis of the tumor cells and an effective blood-brain barrier cross as a single agent or as a combination treatment with TMZ have been demonstrated *in vitro* and *in vivo* (20). Previous studies have demonstrated that Onalespib significantly impairs DNA repair by depleting homologous recombination (HR) proteins such as CHK1 and RAD51, reducing HR repair and increasing glioma stem cell sensitivity to radiation and TMZ (15). It also modulates DDR proteins, including ATM and DNA-PKcs, further compromising repair mechanisms (13). While its impact on MMR proteins is limited, with minor effects on MSH2 and downregulation of MSH4, MSH6, and EXO1, Onalespib’s ability to target multiple DNA repair pathways underscores its potential to overcome treatment resistance (15).

Our study aims to investigate the efficacy and underlying molecular mechanisms of the combining the HSP90 inhibitor Onalespib with external beam radiotherapy in four glioblastoma cell lines *in vitro*, providing a comprehensive model for studying GBM’s genetic diversity. U343 MG and U87 MG, widely used, feature wild-type p53, aiding radiation resistance studies and modeling invasiveness. However, the long-term culturing of these well-established cell lines may have reduced their molecular complexity. In contrast, the patient-derived lines U3013MG and U3024MG retain genetic heterogeneity (22), with sensitivity to certain therapies and exhibiting unique DNA repair defects. This combination supports the development of personalized GBM therapies while ensuring comparability with prior research.

By exploring the combination treatment of Onalespib and radiotherapy, we aim to contribute to the development of more effective therapeutic strategies for GBM and ultimately improve patient outcomes in this challenging disease.

## 2 Materials and methods

### 2.1 Cell lines

The glioblastoma cell lines were purchased from the American Type Culture Collection ATCC (Manassas, VA, United States). U87 MG (HTB-14) cells were grown in Dulbecco’s Modified Eagle’s Medium (DMEM (Biowest, MO, USA)) and was supplemented with 10% Fetal Bovine Serum (FBS, Sigma-Aldrich, Darmstadt, Germany) and 1% antibiotics (100 IU penicillin and 100 μg/ml streptomycin, Biochrom GmbH). The U343 were grown in MEM containing Earle’s salts (Biochrom, Berlin, Germany or Sigma-Aldrich, Darmstadt, Germany) supplemented with 10% FBS (Sigma Aldrich, Darmstadt, Germany), 1% antibiotics (100 IU penicillin and 100 μg/ml streptomycin, (Biochrom GmbH, Berlin, Germany) and 1% sodium pyruvate (Thermo Fisher, Waltham, MA, USA). Both cell lines were grown in an incubator at 37° C and 5% CO_2_. The human patient-derived GBM cell lines U3013MG and U3024MG, were obtained from the HGCC collection (22), and maintained in culture according to HGCC guidelines. Cells were maintained on laminin-coated tissue culture dishes (Primaria, Cat. No. 353802, Corning; laminin Cat. No. L2020, Sigma Aldrich) in a serum-free medium composed of a 1:1 mixture of Neurobasal Medium (Cat. No. 21103-049, Thermo Fisher) and DMEM/F-12, GlutaMAX™ (Cat. No. 10565-018, Thermo Fisher). The medium was supplemented with 10 ng/ml FGF-2 (Cat. No. 100-18B, Peprotech), 10 ng/ml rhEGF (Cat. No. AF-100-15, Peprotech), N-2 (Cat. No. 17502048, Thermo Fisher), and B-27 solution (Cat. No. 17504044, Thermo Fisher).

### 2.2 Drug preparation

Onalespib (AT13387, Selleck Chemicals, Germany) was dissolved in DMSO and stored in aliquots at -20°C. Onalespib was further diluted in complete media for the desired assay concentrations.

### 2.3 Irradiation

For cell viability studies (XTT), migration and multicellular spheroid assays, Proximity Extension analysis and flow cytometry, cells were irradiated 24 h after drug incubation with 225 kV X-rays (X-RAD iR225, Precision X-Ray Inc., North Branford, CT, USA) at a dose-rate of 1.5 Gy/min using an inherent Ba filter (0.8 mm) and an external Cu filter (0.3 mm). For clonogenic survival (24 h after drug incubation), the irradiation was either performed as described above or with an Elekta Versa HD linear accelerator at the Uppsala University Hospital. The X-ray beam was set to 6 MV and the cells were placed at a water-equivalent depth of 10 cm using water-equivalent plastic attenuators. Cells were irradiated using a vertical beam (irradiation from above). The dose rate was approximately 4-5 Gy per minute. All irradiations were performed at room temperature.

### 2.4 XTT assays

The XTT assay was performed to assess the cell viability. U343 MG, U87 MG, U3013MG and U3024MG cells were seeded per well in 96-well plates (VWR, Pennsylvania, USA, laminin-coated for patient-derived cultures) and incubated at 37°C and 5% CO_2_ for 48 h. Cell media was then removed and replaced by fresh media containing 0, 10, 25, 50 and 100 nM of Onalespib, followed by irradiation with 1, 2, 4, or 6 Gy. 72 hours after treatment, an XTT assay (ATCC, Manassa, VA) was performed according to the manufacturer’s protocol. Briefly, XTT activation reagent, XTT reagent and cell media were mixed and 150 μl were added to the 60 inner wells (excluding the outer wells) of the plate, and then the plate was incubated at 37°C and 5% CO_2_ in the dark. The absorbance was measured at 490 and 650 nm in a spectrophotometer 4 hours after incubation (Biorad, iMarkTM Microplate Absorbance Reader). The software used for the measurements was Microplate Manager Software 6 (Biorad). Each measurement was replicated at least six times.

### 2.5 Clonogenic assays

Clonogenic survival assays were performed as described previously (23) to assess the cell’s ability to grow into a colony. In short, 100-4600 U343 cells were seeded in 6-well plates (VWR, Pennsylvania, USA) and incubated at 37°C and 5% CO_2_ for 24 hours. 24 hours later, cells were treated with 2 ml of media-containing Onalespib (5-50 nM). After 24 hours, the cells were irradiated with 2-6 Gy of X-rays and incubated until colonies of more than 50 cells/colony were formed. Then, the medium was removed, followed by washing with cold PBS and the cells were fixated by adding 96% cold ethanol for 20 minutes. and stained with crystal violet (1% solution, Sigma-Aldrich, Darmstad, Germany). Colonies containing more than 50 cells were counted manually and the plating efficiency (PE) and the survival fraction (SF) were calculated. A linear quadratic curve fit (S = exp (−αD − βD 2), where D = radiation dose in Gray, and α and β are fitting parameters) was calculated by using GraphPad Prism 9 software (San Diego, CA, USA).

One-way ANOVA followed by Tukey’s multiple comparison’s test determined significance. Data were expressed as mean SD and p < 0.05 considered to be statistically significant. The number of replicates within each experimental group was 3. Each experiment was repeated at least three times.

### 2.6 Multicellular tumor spheroids

96-well flat bottom plates (VWR, PA, USA) were coated with 50 μl of 1.5% agarose (Sigma Aldrich, Darmstad, Germany) dissolved in PBS (Biowest, MO, USA) according to (24).4500 U343 MG cells and 1500 U87 MG cells were seeded in 200 μl cell media/well and incubated at 37°C and 5% CO_2_ for 72 hours until 3D spheroids formed. Twelve spheroids/group were treated with increasing Onalespib concentrations (50 nM-250 nM). The spheroids were incubated for 24 hours and then irradiated with 2-6 Gy of X-rays. Day 0 was considered to be the treatment day. Media was renewed (100 μl out, 100 μl in) every fourth day. After the treatment, spheroids were followed by photography every 3-4 days for 2 weeks. The images of the cell spheroids were obtained using a 4x magnification with a Canon EOS 700D digital camera (Canon, Tochigi, Japan) mounted on an inverted Nikon Diaphot-TMD microscope (Nikon, Tokyo, Japan). Assuming a spherical spheroid shape, the area of the spheroids was determined using a custom-made macro-command on ImageJ and the volume of the spheroids was calculated. Comparison between groups was performed using one-way ANOVA followed by Tukey’s *post hoc* test. Data were expressed as mean SD and p < 0.05 considered to be statistically significant. The number of replicates within each experimental group was 12. Each experiment was repeated at least three times.

For live/dead cell count, U87 MG, U3013MG, and U3024MG spheroids were treated with 25, 50, and 100 nM Onalespib, as well as 2 or 4 Gy radiation. Three days post-treatment, live/dead cell counts were performed using trypan blue (BioRad) staining according to the manufacturer’s instructions. For the limiting dilution assays, U87 MG cells were trypsinized (accutase was used for patient derived cell lines), and 50, 100, 250, 500, and 1,000 cells were seeded into 96-well round-bottom, ultra-low attachment plates (VWR, PA, USA). Spheroid-forming efficiency was assessed three days later, and the data were analyzed using ELDA software, according to (25).

### 2.7 Migration/proliferation assay

The cell migration and proliferation ability of U343 MG and U87 MG cells was studied using a wound healing assay (also called scratch assay), as previously reported (35). In short, cells were grown at confluence in 6 well plates and a narrow area on the monolayer was scratched off with a p10 pipette tip. Afterwards, wells were washed and incubated with normal cell medium, 5-50 nM Onalespib and radiation of 2-6 Gy. Images from the same scratch location were obtained directly after scratching, 6, 12 and 24 h of incubation using an inverted microscope Nikon Diaphot (Nikon, Japan) mounted with Canon EOS 700D camera (Canon, Tochigi, Japan). Migration distance was measured and analyzed using ImageJ 2.0.0 software (NIH, Bethesda, MD, United States). The experiments were repeated 3 times.

### 2.8 Immunofluorescent biomarker for chromosomal double-strand breaks

The process of preparing slides and quantifying DNA double-strand break (DSB) repair foci was conducted following procedures previously described in (26). Briefly, U343 MG, and U87 MG cells were seeded in 4-well cell culture chamber slides (Nunc A/S, Roskilde, Denmark) to achieve approximately 70% confluency after incubation at 37°C for 24 h. Subsequently, cells were treated with DMSO, and 100 nM Onalespib for 24 h before irradiation with and without 2 and 6 Gy X-rays. Subsequently, samples were washed and replaced with fresh pre-warmed medium. The slides were then incubated at 37°C for 24 h. Afterward, cells underwent a washing step and were fixed with 1X PBS and 99% methanol (-20°C), respectively. Cell membranes were permeabilized with ice-cold acetone (Millipore, Merck, United States) for 10 seconds. Blocking of non-specific proteins was achieved by incubating the cells in 10% FBS PBS for 1 h at room temperature. Following this, the slides were exposed to Rabbit anti53BP1 (1:1000, ab36823, Abcam, Cambridge, United Kingdom) and mouse anti-γH2AX (1:100, JBW301, EMD Millipore Merck Darmstadt, Germany) antibodies overnight at 4°C. The next morning, the slides were incubated with Alexa fluor 555 (1:400, ab150086, Abcam, Cambridge, United Kingdom) and Alexa fluor 488 (1:400, ab150117, Abcam, Cambridge, United Kingdom) for 1 hour in the dark. Nuclei were stained with DAPI (ThermoFisher Scientific, Sweden) in the dark for approximately 2 minutes, followed by washing with 1X PBS and MQ water. The slides were air-dried before mounting with VECTASHIELD^®^ antifade media (part of Maravai LifeSciences, USA). High-resolution images with a 20X NA 0.8 objective were captured using a Zeiss LSM 700 point scanning confocal microscope (Zeiss, Oberkochen, Germany). Foci quantification was performed on maximum intensity projection images using ImageJ software (Fiji Is Just ImageJ). The number of 53BP1 and γH2AX foci were counted for approximately 200 nuclei in each condition.

### 2.9 Flow cytometry

To assess the cell cycle distribution after treatment, flow cytometry was performed. Cells were seeded in T-25 flasks (purchased from VWR) and incubated at 37°C and 5% CO_2_ until confluency was reached. Once confluent, cells were treated with 5 ml of media-containing 500 nM of Onalespib and irradiated with 2 and 4 Gy after 1 hour of drug incubation. After 48 hours, cells were trypsinized followed by washing with PBS and centrifuging (performed twice). Single cell suspensions were prepared by resuspension in PBS. Cold Ethanol was added to fixate the cells. Samples were kept at –20°C for a minimum of one week to ensure cell permeabilization. For flow cytometry analysis, the cells were centrifuged at 1200 rpm for 10 min and washed twice with ice-cold PBS, followed by adding 0.5 mL RNase (100 μg/mL) and 100 μL of PI (50 μg/mL). After 30 min of incubation time (at RT, in darkness) analysis was performed using a CytoFLEX (Beckman Coulter, Krefeld, Germany) flow cytometer. The data analysis and peaks recognition were done by FlowJoTM Software for Windows (Version 10.9 Becton, Dickinson and Company, Oregon, United States).

### 2.10 Western blot analysis

Whole-cell extracts were prepared according to the procedure described in (27). Briefly, the samples were separated using SDS-PAGE and then transferred onto a nitrocellulose membrane (Immobilon-P Transfer membrane, Millipore, Merck) through wet blotting. The membrane was blocked for 1 hour in PBS containing 5% BSA and incubated overnight at 4°C with a monoclonal p21 antibody (1:1000, ab109520, Abcam, Cambridge), an anti-γH2AX antibody (1:2000, ab11174, Abcam, Cambridge), and an anti-GAPDH antibody (1:500,000, ab8245, Abcam, Cambridge) as a protein loading control.

After three washes with PBS-Tween (1%), a secondary antibody conjugated with Horseradish Peroxidase specific to the primary antibody species was added for 1 hour at room temperature. This was followed by another three washing steps with PBS-Tween (1%). The immunoreactive bands were then visualized using an Amersham ImageQuant 800FL imaging system (Cytiva Life Science, Uppsala, Sweden) after applying an electrochemiluminescent reagent (Immobilon, Millipore). Uncropped Western blot membranes are shown in **Supplementary Figure 5**.

### 2.11 Proteomic analysis: proximity extension assay

U343 MG cell culture lysates were analyzed with Olinks Proximity Extension Assay using the Oncology II panel (v.7004, Olink Biosciences, Uppsala, Sweden), measuring expression of 96 proteins. Lysates taken at 24 h post-treatment of 500nM Onalespib or X-ray irradiation of 4 Gy or the combination of the two. Protein levels were expressed as normalized protein expression (NPX) on a log2-scale. Values below limit of detection (LOD) were truncated at the LOD. No values were above the upper limit of quantification.

All data analysis was performed with R (v4.3.1). In order to analyze expression signatures between treatments, hierarchical clustering was performed using the hclust function.

To identify important proteins, the standard deviation of each assay was used. A large standard deviation (big differences between treatments) corresponded to a high rank. This was performed on NPX values, normalized (by subtraction) to the control sample of the corresponding treatment, with the std function. Functional ontology analysis of the highly ranked proteins was performed using the clusterProfiler (v 4.0) package (28, 29), and the Reactome pathway knowledgebase (v87) as reference (30).

### 2.12 Statistical analysis, synergy analysis and tumor spheroid doubling time

The experimental data were analyzed using Microsoft Office Excel for Mac Version 16.8, and graphs were generated using GraphPad Prism 10 for Mac OS X. Statistical analysis of the viability, proliferation and migration assays was conducted using one-way ANOVA with Tukey’s post-test in GraphPad Prism 9. Statistical analysis of cell cycle distribution was conducted using two-way ANOVA with Tukey’s post-test in R (v4.3.1), using an interaction term between the cell cycle and treatment factors, independent of cell line effects (fraction ~ cycle * treatment + cell line), and the within treatment groups contrasts were compared in the *post-hoc* analysis. A p-value of ≤ 0.05 was considered statistically significant. The results are presented as means ± standard deviation (SD).

Synergy calculations for proliferation, clonogenic survival and migration assay data were performed using the SynergyFinder website (https://synergyfinder.org, accessed in February 2024). This analysis generated dose-response curves and provided Loewe synergy scores.

To evaluate the combined effects of Onalespib and external beam radiotherapy on multicellular tumor spheroid growth, the Loewe method was employed on day 14 of the experiment. A Loewe score ≥10 was considered synergistic, <10>-10 additive, and ≤ -10 antagonistic.

The tumor doubling time was determined using a modified Schwartz formula, expressed as follows: tumor doubling time = [ln2 × ΔT]/[ln (X_2_/X1)], where X_1_ represents the tumor size at the initial treatment day, X_2_ represents the spheroid size at day 14 and ΔT denotes the time (in days) between the two measurements.

## 3 Results

### 3.1 Synergistic anticancer effects of combining Onalespib with radiotherapy on metabolic activity and cell viability

To determine cell viability of glioblastoma cells after exposure to various doses of the HSP90 inhibitor Onalespib and external radiation, metabolic activity was measured using XTT assay. The established glioblastoma cell lines U343 MG, U87 MG as well as the patient-derived glioblastoma lines U3013MG and U3024MG were exposed to Onalespib treatment at multiple doses followed by the application of radiation therapy 24 h after drug incubation, and absorbance measurement 72 h after drug treatment.

**Figure 1:**
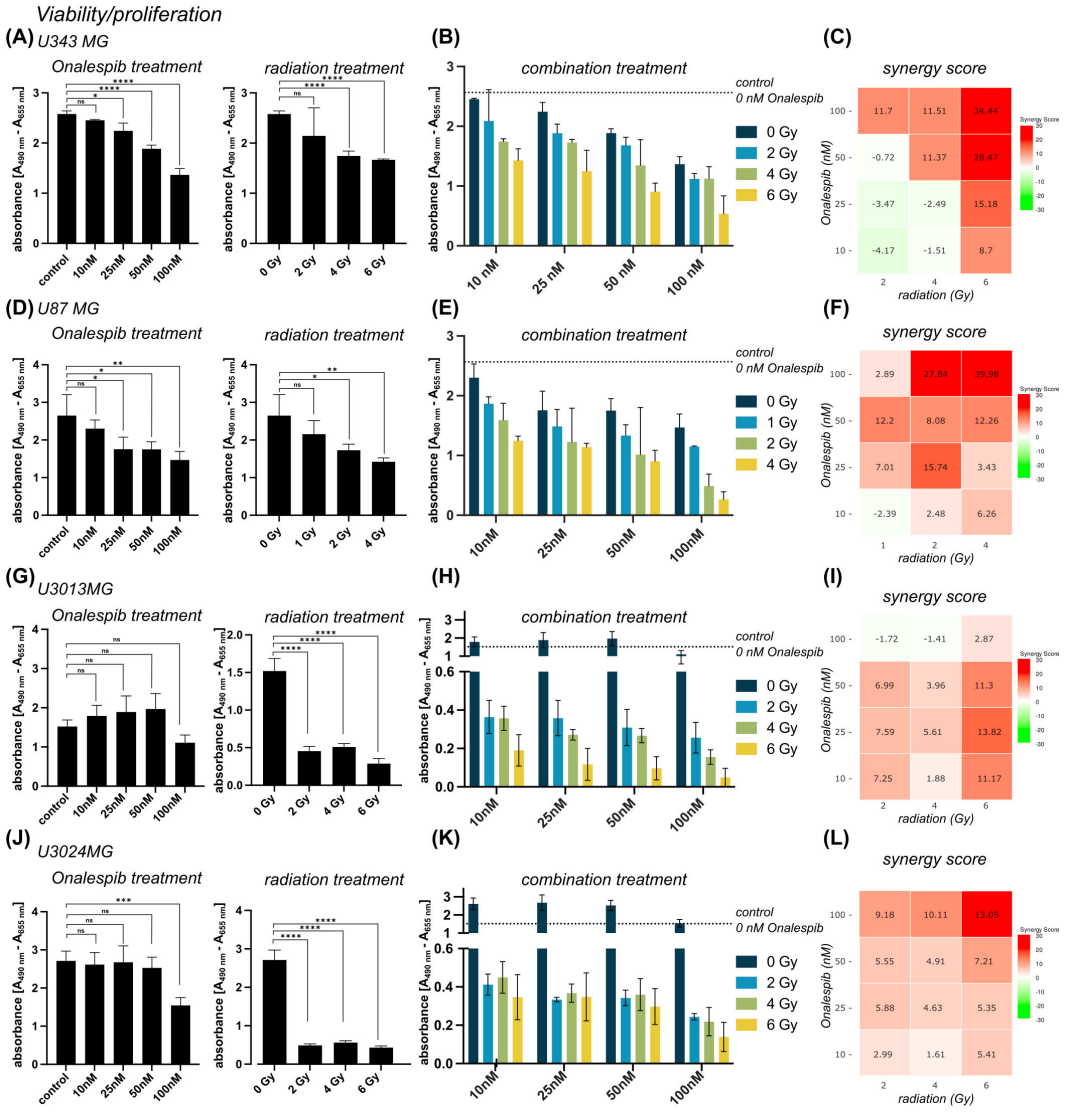
Viability of U343 MG **(A-C)**, U87 MG **(D-F)** U3013MG **(G-I)** and U3014MG **(J-L)** determined by XTT assay. **(A)** Viability (absorbance) after 0, 10, 25, 50 and 100 nM Onalespib treatment (left) and after radiotherapy with 0, 2, 4, 6 Gy (right) **(B)** combination effect of Onalespib and radiotherapy **(C)** LOEWE synergy scores **(D)** Viability (absorbance) after 0, 10, 25, 50 and 100 nM Onalespib treatment (left) and after radiotherapy with 0, 1, 2, 4 Gy (right) Right) **(E)** combination effect of Onalespib and radiotherapy **(F)** LOEWE synergy scores. **(G)** Viability (absorbance) after 0, 10, 25, 50 and 100 nM Onalespib treatment (left) and after radiotherapy with 0, 2, 4, 6 Gy (right) **(H)** combination effect of Onalespib and radiotherapy **(I)** LOEWE synergy scores **(J)** Viability (absorbance) after 0, 10, 25, 50 and 100 nM Onalespib treatment (left) and after radiotherapy with 0, 2, 4, 6 Gy (right) **(K)** combination effect of Onalespib and radiotherapy **(L)** LOEWE synergy scores. Data plotted as means ± standard deviation. One-way ANOVA with Tukey’s post-test ns (not significant), *(p < 0.05), **(p < 0.01), ***(p < 0.001) and ****(p < 0.0001).

Results from both U343 MG and U87 MG revealed a significant dose-dependent decrease in cell viability and proliferation in following drug and radiation monotherapy (**Figures 1A, B, D, E**). Both glioblastoma cell models demonstrated a similar response to Onalespib treatment, e.g., inhibiting viability/proliferation by 47 and 44.5%, respectively, at a dose of 100 nM. U87 MG presented more sensitive to radiation, 46.4% survived a radiation dose of 4 Gy, while 68% of U343 MG cells were viable after the same dose. Furthermore, additional exposure of 25 nM resulted in a 13% and 24% reduction in the viability of U343 MG and U87 MG cells, respectively. In contrast, the patient-derived cell lines U3013MG and U3024MG showed no significant reduction in viability at low Onalespib concentrations, with only 100 nM causing a notable decrease (**Figures 1G, J**, left). However, both cell lines were highly sensitive to radiation, with 2 Gy reducing viability by 70.1% in U3013MG and 82% in U3024MG (**Figures 1G, J**, right).

The combined treatment was more effective for all established and patient-derived cell lines with the highest inhibition at the higher doses (**Figures 1B, C, E, F, H, K**). Synergistic combination effects, as evidenced by Loewe synergy values > 10, were observed at all drug doses >10 nM and 6 Gy of radiation. At lower radiation doses additive effects were observed except for drug concentrations ≥ 50 nM for U343 MG (**Figure 1C**). U87 MG demonstrated a similar pattern, with the highest synergistic values recorded at higher concentrations. However, synergistic effects were also observed at lower drug and radiation doses (**Figure 1F**). In patient-derived cell lines, U3013MG showed synergy at 10nM, 25 nM and 50 nM combined with 6 Gy (**Figure 1I**), while U3024MG exhibited synergy at 100 nM and 4Gy as well as 6 Gy (**Figure 1L**).

### 3.2 Synergistic anticancer effects of combining Onalespib with radiotherapy on clonogenicity (2D) and multicellular tumor spheroid growth (3D)

**Figure 2:**
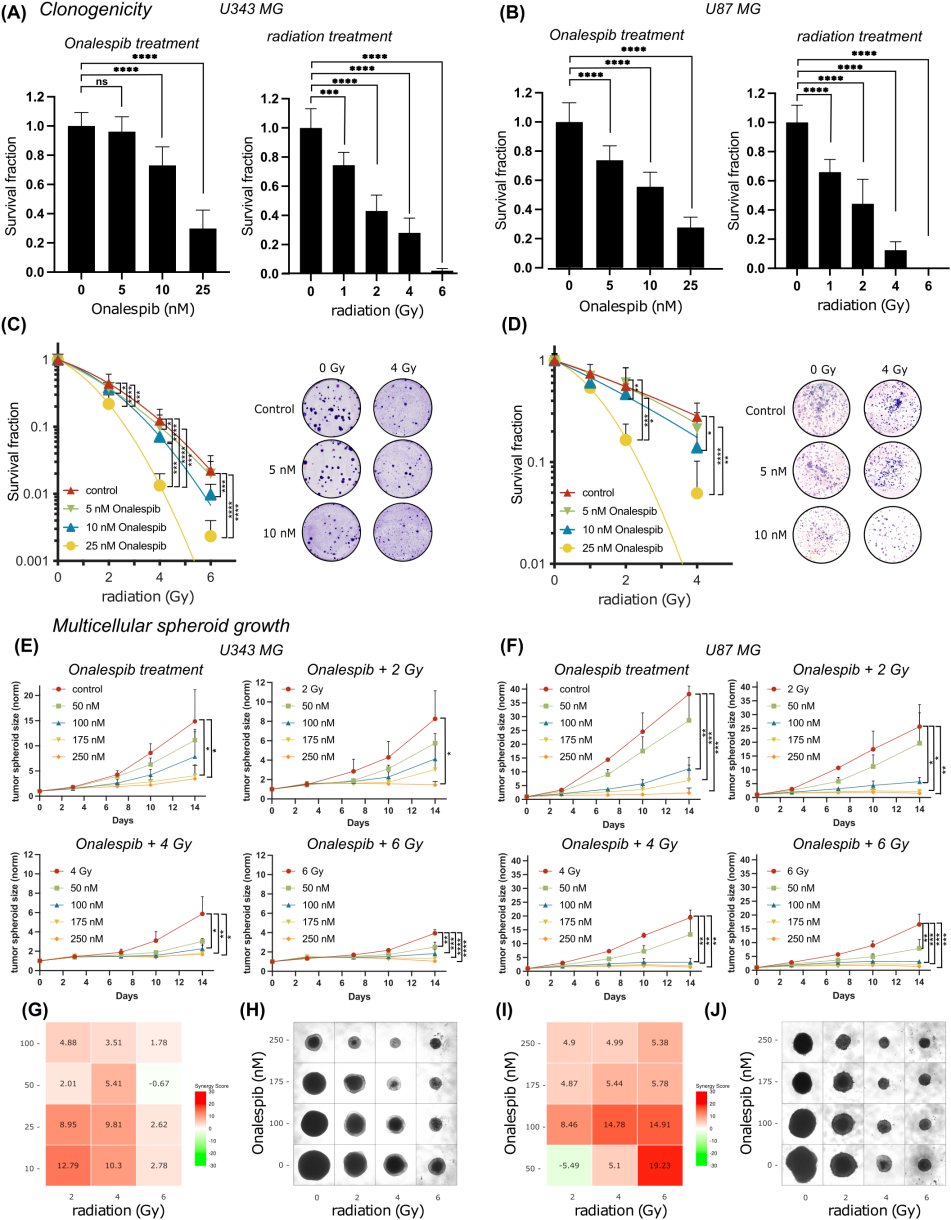
Colony formation and multicellular spheroid growth of U343 MG and U87 MG glioblastoma cells. Survival fraction of Onalespib and radiation treated U343 MG **(A)** and U87 MG **(B)** Survival fraction of Onalespib and radiation combination treatment of U343 MG **(C)** and U87 MG **(D)**. Representative images of the colonies of the monotreatments and the combined treatments of Onalespib and radiation. Onalespib monotherapy and combination therapy with radiation in 3D spheroid model of U343 MG **(E)** and U87 MG **(F)**. Graphs display the normalized spheroid volume (mm3) over time, (means ± standard deviation, n ≥ 3). LOEWE synergy scores for U343 MG **(G)** and U87 MG **(I)**. Representative images of the U343 MG and U87 MG multicellular tumor spheroids at the endpoint of the assay are shown in **(H, J)**, respectively. Data plotted as means ± standard deviation, n ≥ 3. One-way ANOVA with Tukey’s post-test ns (not significant), *(p < 0.05), **(p < 0.01), ***(p < 0.001) and ****(p < 0.0001).

To further evaluate the effectiveness of combining radiation with Onalespib in glioblastoma clonogenic assay were performed (**Figures 2A–D**). Both radiation treatment and Onalespib treatment decreased cell survival in a concentration-dependent manner. Significant clonogenicity reduction was noted at 1, 2, 4, and 6 Gy. In line with the viability assays (see above), U87 MG showed an increased radiosensitivity compared to U343 MG. Further, monotreatment with 10 and 25 nM Onalespib significantly decreased to colony formation ability or U343 MG and 5,10 and 25 nM for U87 MG compared to DMSO-treated control samples (**Figures 2A, B**). A complete loss of colony formation was observed for 50 nM of Onalespib regardless of the delivered radiation dose (data not shown). Combination treatment of Onalespib and radiation decreased the clonogenicity even more, most pronounced at the highest drug and radiation doses (**Figures 2C, D**). However, even at low radiation doses, the combination treatment with 25nM Onalespib was extremely potent. A clinically relevant radiation dose of 2 Gy in combination with 25nM Onalespib reduced the colony formation by 78.2% and 83.5% for U343 MG and U87 MG, respectively.

To mimic *in vivo* conditions, the efficacy of the drug and radiation treatment was tested in multicellular 3D tumor spheroid model (**Figures 2E, F**). Interestingly, the U343 MG and U87 MG glioblastoma spheroids exhibited less sensitivity compared to the previously evaluated 2D models. In line with the 2D models however, combined treatment with Onalespib and radiation resulted in concentration dependent additional inhibition of growth compared to individual treatments. The *in vitro* tumor spheroid doubling times for the different Onalespib treatments and radiation doses are summarized in **Table 1**. Untreated U343 MG and U87 MG tumor spheroids exhibited doubling times of 3.34 and 2.47 days, resulting in a volumetric increase of 1386% and 3720% after 14 days, respectively. Treatment with 250 nM Onalespib and 6 Gy radiation was able to significantly reduce proliferation and increase the doubling time to 67.31 and 26.23 days, respectively, corresponding to a volume increase of 14% and 41%.

**Table 1:** U343 MG and U87 MG tumor spheroid doubling time (TDT, days) and tumor spheroid volume (V) increase (%) after treatment with Onalespib (nM) and radiotherapy (Gy) on day 14.

	0 nM		50 nM		100 nM		175 nM		250 nM	
U343 MG	TDT (days)	V (%)	TDT (days)	V (%)	TDT (days)	V (%)	TDT (days)	V (%)	TDT (days)	V (%)
0 Gy	3.34	1386	3.74	1013	4.05	823	5.47	420	7.26	246
2 Gy	4.17	770	5.15	474	6.35	313	8.13	203	24.18	45
4 Gy	5.09	487	8.18	201	11.01	127	17.01	70	33.76	31
6 Gy	6.58	294	9.76	152	14.88	83	32.51	32	67.31	14
U87 MG	TDT (days)	V (%)	TDT (days)	V (%)	TDT (days)	V (%)	TDT (days)	V (%)	TDT (days)	V (%)
0 Gy	2.47	3720	2.68	2770	4.14	780	5.47	420	9.43	160
2 Gy	2.78	2460	2.83	2320	5.18	470	11.36	121	18.92	61
4 Gy	3.03	1850	3.48	1232	7.61	227	13.39	96	19.98	57
6 Gy	3.21	1560	4.35	693	7.77	219	15.33	80	26.23	41

Synergy calculations performed 14 days post treatment exposure, where a LOEWE synergy score of >10 indicated synergy, showed potentiating synergistic effects for several combinations of Onalespib (10 and 25 nM) and 4 and 6 Gy. This observation is also reflected in the glioblastoma spheroid images in **Figures 2H, J**.

To further characterize the multicellular tumor spheroids, we quantified live cells by labeling dead cells with trypan blue staining three days after treatment with monotherapies of 25, 50, and 100 nM Onalespib and 2 or 4 Gy radiation on U87 MG, as well as patient-derived U3013MG and U3024MG tumor cell spheroids. No significant differences in spheroid size were observed across treatments at that timepoint. Interestingly, although the spheroid sizes remained comparable, the live cell/dead cell count within the spheroids varied. A strong correlation was observed between higher treatment doses and an increase in the dead cell population, **Supplementary Figure 2A**.

Additionally, we assessed spheroid-forming efficiency through limiting dilution assays using U87 MG, U3013MG, and U3024MG cells. These cell lines were treated with 25, 50, and 100 nM Onalespib, 2 or 4 Gy radiation, and combination therapies. All untreated controls of the three cell lines efficiently formed spheroids. However, increasing doses of both the drug and radiation led to a dose-dependent reduction in spheroid-forming capacity, **Supplementary Figure 2B**.

### 3.3 Interrupted migration potential of glioblastoma cells treated with Onalespib and radiotherapy

**Figure 3:**
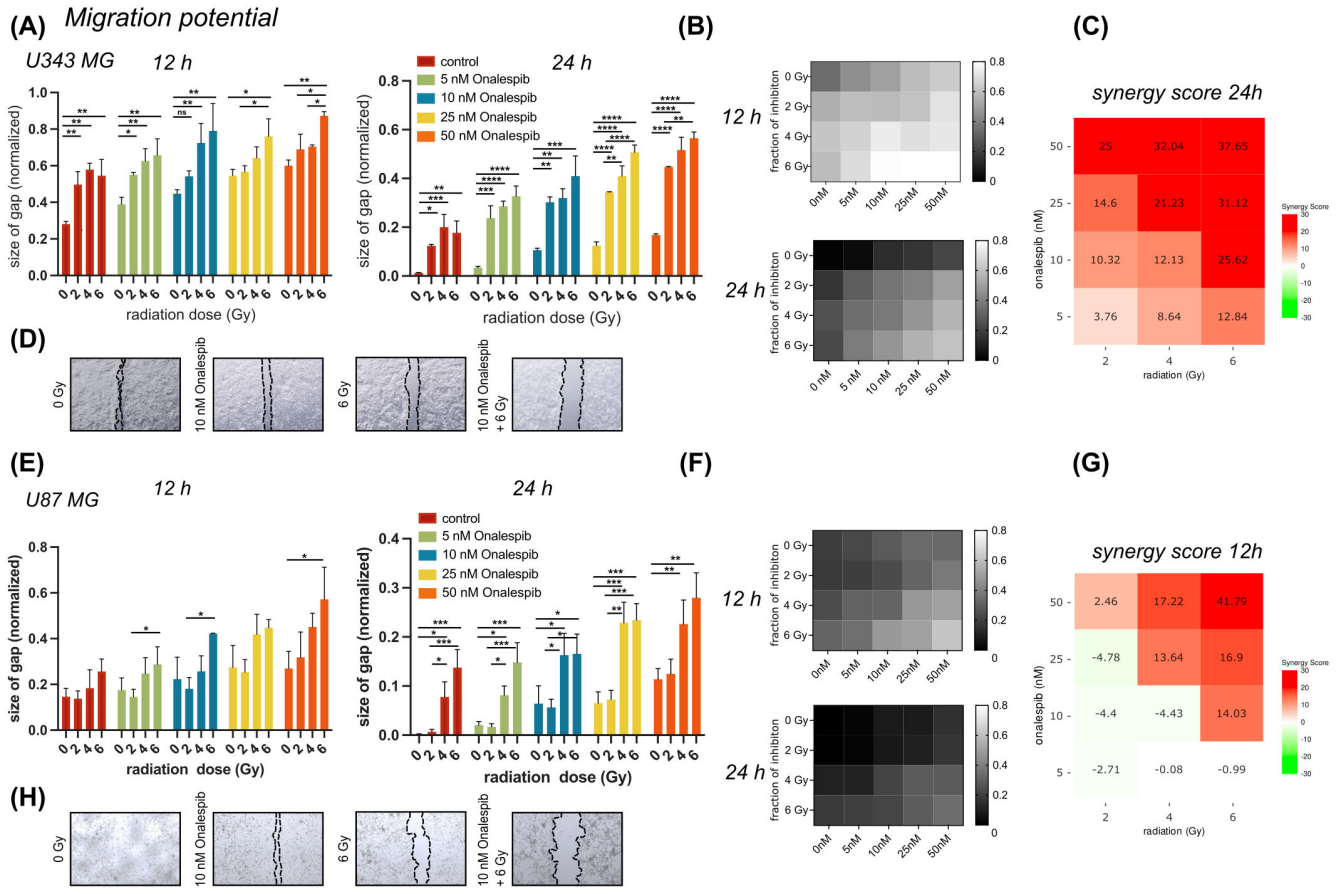
Wound healing/migration potential of U343 MG and U87 MG glioblastoma cells. **(A)** Effect of 0, 5,10, 25 or 50 nM of Onalespib combined with 0, 2, 4 or 6 Gy on U343 MG after 12 and 24 hours. **(B)** Heat map of mono- and combination treated U343 MG cells after 12 and 24 h. **(C)** 24 h U343 MG LOEWE synergy scores. **(D)** Representative images of scratched area. **(E)** Effect of 0, 5,10, 25 or 50 nM of Onalespib combined with 0, 2, 4 or 6 Gy on U87 MG after 12 and 24 hours. **(F)** Heat map of mono- and combination treated U343 MG cells after 12 and 24 h. **(G)** 12 h U87 MG LOEWE synergy scores. **(H)** Representative images of scratched area. Data plotted as means ± standard deviation, n ≥ 3. One-way ANOVA with Tukey’s post-test ns (not significant), *(p < 0.05), **(p < 0.01), ***(p < 0.001) and ****(p < 0.0001).

Wound healing assays (scratch assays) were performed to explore the impact of Onalespib and radiation treatment as well as their combination on the migratory capacity of U343 MG and U87 MG cells (**Figure 3**).

In both glioblastoma cell lines, monotreatment with Onalespib as well as radiation resulted in a concentration dependent reduction in cell migration compared to untreated control cells (**Figures 3A, E**). Generally, U343 MG cells (**Figures 3A, B**) migrated slightly slower as U87 MG (**Figures 3E, F**). Representative images of the U343 MG and U87 MG after the mono- and combination therapies are shown in **Figures 3D, H**, respectively. At the 12 h post treatment time point U343 MG had migrated and closed the wound by 72% while U87 MG had covered 86% of the induced wound. A radiation dose of 2 Gy reduced the migration potential significantly in U343 MG cells and augmented with increasing drug concentrations. Synergy scores for all drug and radiotherapy combinations are summarized in **Figures 3C, G** and **Supplementary Figure 1**. Surprisingly, 2 Gy had no significant effect on U87 MG cells measured at 12 and 24 h. The Onalespib and radiation combination effect was most clear in the higher combination treatment groups. While untreated control cells had closed the gap at 24 h, U343 MG cells treated with 50 nM Onalespib and 6 Gy radiation, had only migrated 44% and U87 MG 77% of that distance.

### 3.4 Accumulation of DNA double-strand breaks glioblastoma cells subjected to Onalespib and radiation combination treatments

**Figure 4:**
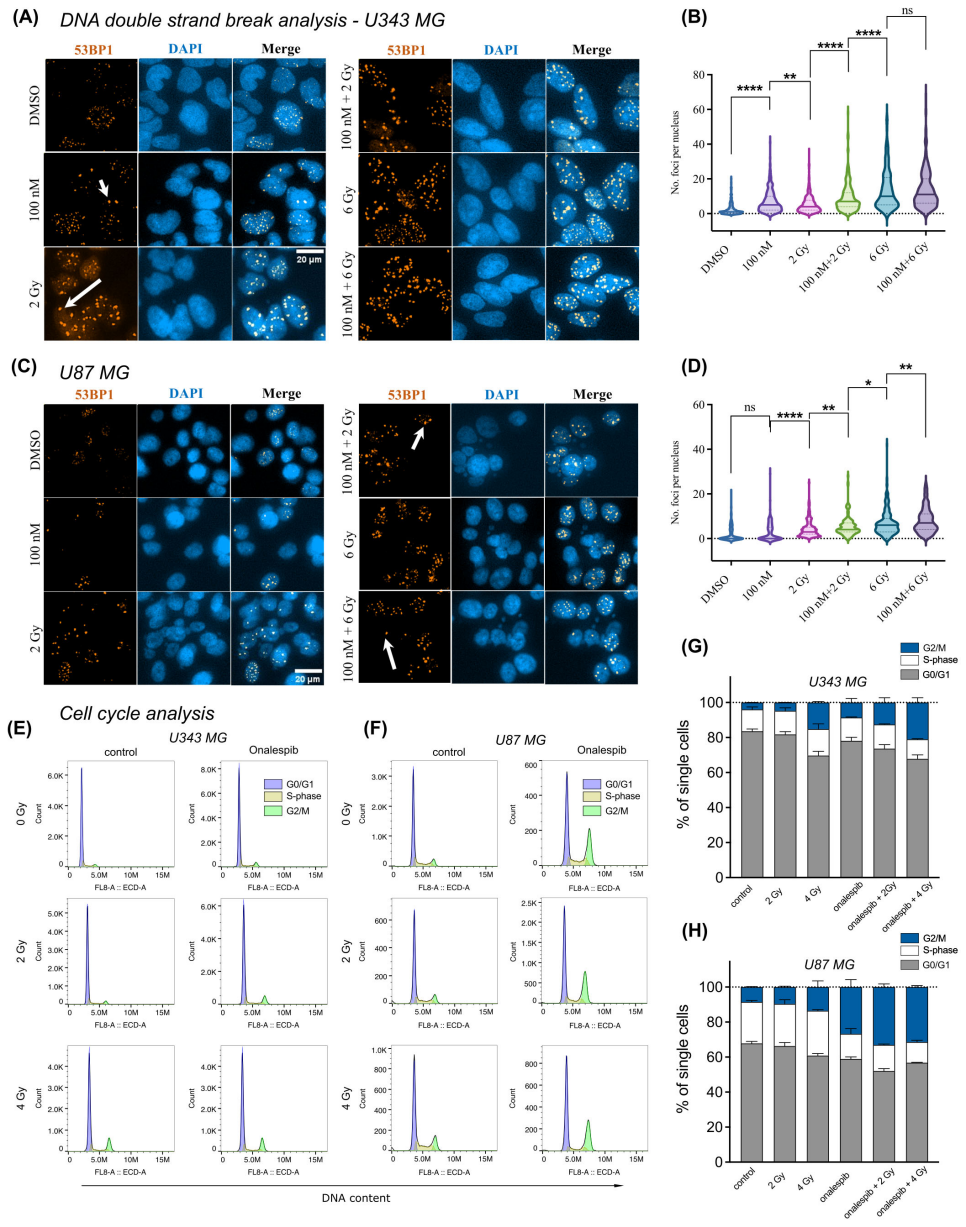
Distribution of 53BP1 foci analysis of U343 MG and U87 MG cells. **(A)** Confocal microscopy images of U343 MG cells treated with 100 nM Onalespib and 2 and 6 Gy radiation. Arrows indicate representative instances of counted 53PP1 foci. **(B)** Violin plots of U343 MG, number of 53BP1 foci per cell. **(C)** Confocal microscopy images of U87 MG cells treated with 100 nM Onalespib and 2 and 6 Gy radiation. Arrows indicate representative instances of counted 53BP1 foci. **(D)** Violin plots of U87 MG, number of 53BP1 foci per cells. Cell cycle analysis by flow cytometry of **(E)** U343 MG and **(F)** U87 MG cells 48 h after exposure of a single dose of 2 Gy, 4 Gy radiation and/or Onalespib, representative histograms. Average cell cycle distribution of **(G)** U343 MG and **(H)** U87 MG. Data plotted as means ± standard deviation, n = 2. ns (not significant), *(p < 0.05), **(p < 0.01) and ****(p < 0.0001).

We assessed DDR by measuring DNA double-strand breaks (DSBs) in U343 MG and U87 MG glioblastoma cells treated with Onalespib, radiation, or their combination using confocal microscopy (**Figures 4A–C**). The number of 53BP1 and γH2AX foci, both markers for DSBs, were counted in the cell nuclei. In both glioblastoma cell lines, untreated cells exhibited a low number of 53BP1 and γH2AX foci per nucleus, 2.5 53BP1 foci/cell and 0.4 γH2AX foci/cell for U343 MG and 2 53BP1foci/cell and 0.3 γH2AX foci/cell for U87 MG (**Figure 4**, **Supplementary Figures 3A–F**).

In U343 MG cells, both Onalespib and 2 Gy radiation monotherapies significantly increased the number of 53BP1 and γH2AX foci, with Onalespib alone inducing more foci than 2 Gy of radiation alone. The combination of 2 Gy radiation and Onalespib further increased the number of DNA damage foci. Increasing the radiation dose to 6 Gy in combination with Onalespib dramatically elevated 53BP1 and γH2AX foci expression, indicating extensive DSB accumulation and reduced repair efficiency. Notably, cells treated with 6 Gy radiation, both alone and in combination with Onalespib, exhibited a high number of foci with about 14 53BP1 foci/cell, reflecting unrepaired DSBs and an impaired repair capacity (**Figures 4A, B**). The minor difference observed between the group treated with 6 Gy alone and the group treated with a combination of 6 Gy and Onalespib may be attributed to the U343 MG cells reaching their maximum threshold for DNA repair capacity, as reflected in both 53BP1 and γH2AX foci. When cells are exposed to high levels of radiation, their ability to repair damaged DNA can become overwhelmed.

In U87 MG cells, Onalespib monotherapy led to a minor increase in both γH2AX and 53BP1 foci. However, 2 Gy radiation significantly elevated the number of the foci compared to Onalespib treatment alone. The combination of 2 Gy radiation and Onalespib further enhanced the DSB repair response. Increasing the radiation dose to 6 Gy combined with Onalespib resulted in a dramatic increase in remaining 53BP1 foci (8 foci/cell), indicating a failure of U87 MG cells to effectively repair the extensive DNA damage caused by the combination therapy (**Figures 4C, D**). While generally lower γH2AX foci counts were observed across all treatment groups, the differences closely mirrored the variations in 53BP1 foci between groups in both cell lines. Representative images of the co-expression of 53BP1 and γH2AX are shown in **Supplementary Figure 3** for both U343 MG and U87 MG cells. Both proteins were simultaneously activated by radiation and Onalespib, with foci appearing in close proximity within the nucleus, suggesting potential co-localization. However, some foci, mainly 53BP1, were also found in distinct nuclear regions.

To further substantiate the findings from confocal microscopy, we performed Western blot analysis of U343 MG cells to evaluate γH2AX levels in the control, radiotherapy, Onalespib, and combination treatment groups. The results, displayed in **Supplementary Figure 3F** (right), revealed that, as expected, γH2AX expression was significantly increased in the radiotherapy-treated group, indicating pronounced DNA damage. Treatment with Onalespib alone led to a rise in γH2AX levels compared to the control. The combination therapy also resulted in elevated γH2AX levels, albeit to a slightly lesser extent than the radiotherapy group alone.

Comparing U343 MG and U87 MG cells’ DSB repair capacity in response to Onalespib and X-rays mono and combination therapy showed that, in both cell lines, Onalespib effectively decreased the cell DSB repair capacity in combinational treatment groups via inducing complex DSBs.

### 3.5 Alterations in cell cycle distribution of glioblastoma cells subjected to Onalespib and radiation combination treatments

We employed flow cytometric analysis to investigate alterations in cell cycle distribution of the cell lines U343 MG and U87 MG after exposure (48 h) to 100 nM Onalespib, 2 and 4 Gy radiation and their combinations (**Figures 4E–H**). Our findings show distinct changes in cell cycle phases compared to untreated controls. Specifically, a 4 Gy radiation dose reduced the percentage of cells in the G0/G1 phase from initially 83.5% to 70% in U343 MG cells and from 68% to 60% in U87 MG cells. At the same time, there was an increase in the number of cells in the G2/M phase for both investigated glioma cell lines. Combination treatment with Onalespib enhanced this effect, resulting in 21% of U343 MG cells and 31% of U87 MG cells being arrested in the G2/M phase. Additionally, we observed that combination of Onalespib and radiotherapy treatment reduced the percentage of cells in the S-phase compared to the untreated control samples, with the most pronounced effect seen in U87 MG cells (**Figure 4H**), but not in U343 MG cells. These changes were statistically significant in a two-way ANOVA model (**Supplementary Table 1**).

We also studied p21 expression by Western blotting (**Supplementary Figure 3F** left), which confirmed the findings from the cell cycle flow analysis and aligned with the PEA analysis presented in the next paragraph. HSP90 inhibition by Onalespib suppressed the expression of CDKN1A (p21), a crucial regulator of cell cycle progression at G1 and S phase. However, both radiotherapy alone and in combination with Onalespib resulted in increased p21 expression, suggesting the initiation of cell cycle arrest following DNA damage and activation of cell death pathways.

### 3.6 Proteomic analysis of glioblastoma cells subjected to Onalespib and radiation combination treatments

**Figure 5:**
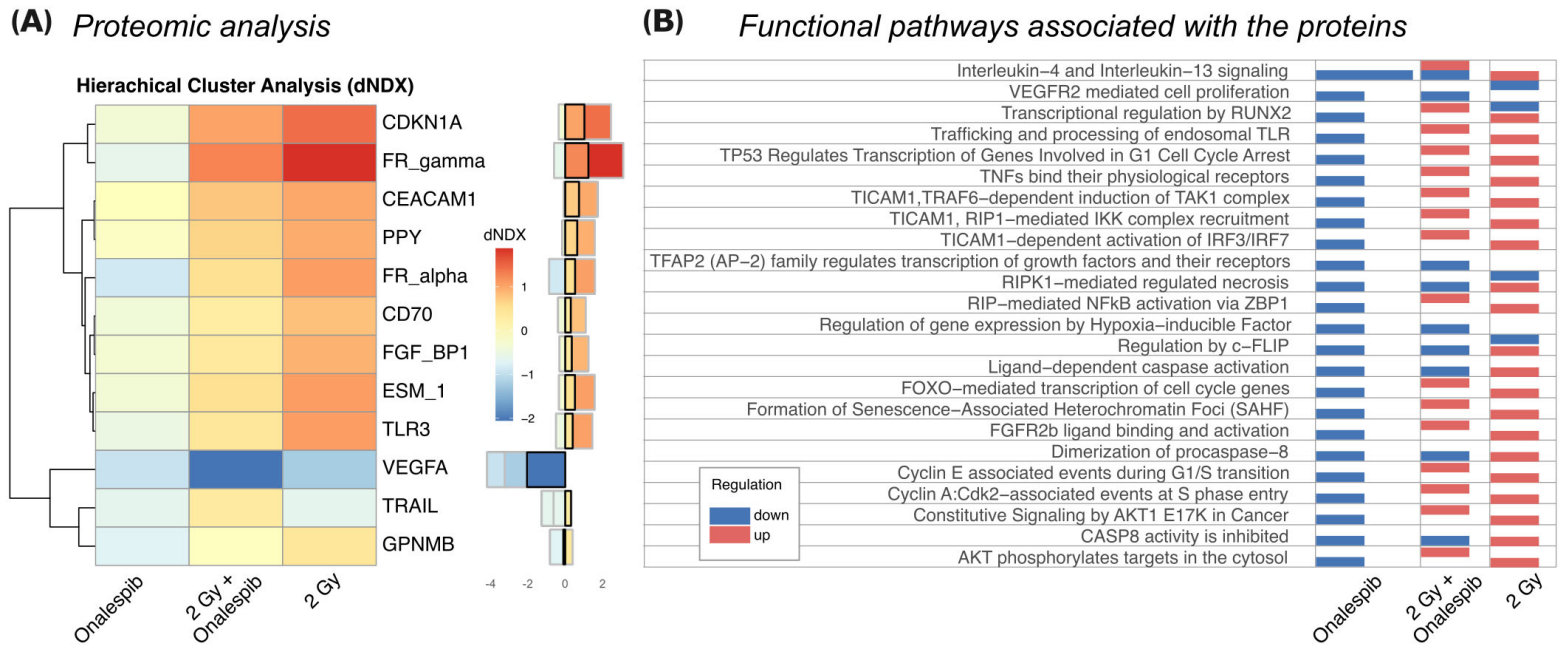
**(A)** The 12 most differentially expressed proteins between treatments (SD>0.5). Hierarchical clustering analysis illustrates the most prominent alterations in protein expression levels observed in U343 MG cells treated with radiation, Onalespib, and their combination, relative to untreated control cells. The difference in log(expression) to control (dNDX) is indicated, with positive values highlighted in red, indicating higher expression compared to control, and negative values shown in blue, indicating lower expression. Middle) Absolute dNDX values for each treatment group relative to the control are depicted using the same color scale as in panel **(A)**. The black square designates the combination treatment group. **(B)** Functional analysis indicating the main ontological pathways where the differentially expressed proteins are involved, and the overall direction of protein regulation. Note that up and down-regulation of specific proteins do not necessarily imply induction or suppression of the functional pathway which the protein is involved in.

The proteomic analysis conducted on U343 MG cells treated with Onalespib, radiation, and their combination revealed significant alterations in protein expression profiles. Hierarchical cluster analysis, shown in **Figure 5** and **Supplementary Figure 4**, depicted distinct differences in protein expression among the treatment groups compared to control cells. Notably, Onalespib treatment primarily led to the downregulation of most tested proteins, while radiotherapy exhibited an overall inducing effect on protein expression. Combination therapy functionally resembled radiation therapy, except for proteins involved in necrosis, c-Flip, caspase and procaspase activity which were upregulated in comparison to radiation therapy alone.

Of particular interest were the changes in protein expression associated with cancer development, including pathways related to growth signaling, replicative potential, angiogenesis, metastasis, invasion, and resistance to cell death. In agreement with the Western blot analysis, Onalespib-mediated HSP90 inhibition decreased CDKN1A (p21) expression. However, when radiotherapy was administered, either on its own or together with Onalespib, there was an increase in p21 levels, indicating the induction of cell cycle arrest due to DNA damage and the activation of cell death mechanisms.

Additionally, FR-gamma (Folate receptor 3, FOLR3), a folate receptor essential for DNA synthesis, was suppressed by Onalespib. On the other hand, radiotherapy strongly induced its expression, potentially indicating an increased demand for folate during DNA damage response processes. However, the expression level within the combination treatment group was lower than with radiotherapy alone, suggesting that HSP90 downregulation by Onalespib reduces folate uptake. Folic acid can mitigate radiation-induced DNA damage by enhancing DNA synthesis and repair, as well as functioning as a radical scavenger. Similarly, FR_alpha (Folate receptor 1, FOLR1) was upregulated after exposure to radiation but decreased under HSP90 inhibition. This decrease may be beneficial, as elevated FOLR1 levels correlate with aggressive tumor characteristics, diminished response to chemoradiotherapy, and poorer overall survival rates.

VEGFA, a key regulator of angiogenesis, was strongly downregulated by HSP90 inhibition and further suppressed by radiotherapy. In line with these results, the combination treatment markedly decreased its expression, indicating a potential inhibition of tumor vascularization and growth.

Furthermore, TRAIL, a cytokine inducing apoptosis, was reduced by both HSP90 inhibition and radiotherapy individually. Apart from apoptotic cell death, TRAIL can mediate a programmed form of caspase-independent cell death known as necroptosis. Combination treatment significantly upregulated TRAIL expression, suggesting enhanced activation of tumor cell death mechanisms.

The functional analysis of the differentially expressed proteins (**Figure 5B**) identifies ontological pathways relevant to cancer development and treatment response which the proteins are involved in. Downregulation of proteins involved in growth factor-mediated signaling might indicate inhibition of cell proliferation and survival pathways, potentially impeding tumor progression. Conversely, upregulation of proteins involved in IL-4 and IL-13 signaling might indicate immune response modulation, possibly enhancing anti-tumor immunity or altering the tumor microenvironment. Induction of proteins involved in p53-induced cell cycle arrest pathways can imply activation of DNA damage response mechanisms, likely contributing to cell cycle arrest and inhibition of tumor cell proliferation. Additionally, upregulation of proteins involved in caspase activation suggests increased apoptotic cell death, potentially enhancing the anti-tumor effects of the treatments.

## 4 Discussion

GBM is characterized by HSP90 overexpression, aggressive growth, and poor prognosis (31). In cancer cells, the mechanisms of the HSP90 chaperone system differ significantly from those in normal cells. The rapid proliferation rate and reduced quality control in protein synthesis lead to increased and constant cellular stress. HSP90 stabilization has been developed as a coping mechanism, and HSP90 expression is 2- to 10-fold higher in cancer cells compared to normal cells, aiding in cell survival and function during tumorigenesis (20, 32). There is a connection between proliferation rate and expression level, and therefore high expression of HSP90 is associated with a poor prognosis in clinical treatment.

Due to the high innate resistance of GBM to standard treatments, it is crucial to find new agents that re-sensitize cancer cells to improve treatment efficacy. Combination therapy can enhance efficacy, reduce toxicity, and lower the incidences of drug resistance by exploiting the synergy of action (33). To date, the combination of HSP90 inhibitors with chemotherapy (27), targeted agents (34, 35), or immunotherapy (36) has demonstrated enhanced antitumor effects, summarized in (37).

In this study, we investigate the efficacy of Onalespib in combination with radiotherapy in two patient-derived glioblastoma cell lines U3013MG and U3024MG as well as the established cell lines U343 MG and U87 MG. Onalespib targets HSP90, overexpressed in cancer cells, suggesting selective targeting of tumor cells while sparing healthy brain tissue. Its ability to cross the blood-brain barrier and achieve higher concentrations in brain tissue further supports its potential in brain cancer treatment.

While early clinical trials showed a favorable toxicity profile, with mild adverse events such as diarrhea, fatigue, and nausea, they did not focus on neurotoxicity or radiation therapy interactions (19, 21, 38, 39).

Our findings indicate that the combination of Onalespib with radiotherapy improves anti-tumor effects by decreasing cell viability, proliferation, and clonogenicity in the assessed cell lines grown in monolayer cell culture in a concentration-dependent manner (**Figures 1**, **2**).

Further, multicellular tumor spheroid models, which mimic the *in vivo* microenvironment, such as hypoxic areas within avascular tumors, offer a valuable platform for pre-clinical drug and radiotherapy testing. This is a highly relevant model system in these investigations since lack of oxygen is associated with resistance to radiotherapy. HSP90 is upregulated in GBM spheroid models facilitating stem-like characteristics such as self-renewal, differentiation, tumorigenicity, and drug resistance. Our study shows that GBM tumor spheroids were more resistant to treatment, requiring higher concentrations compared to 2D experiments. However, the proliferation and doubling time of both U87 MG and U343 MG tumor spheroids were significantly reduced by Onalespib monotreatment, with combination treatment showing the most potent effects. Additionally, limiting dilution analysis and live/dead staining indicated a concentration-dependent decrease in spheroid formation capacity and an increased percentage of dead cells within the spheroids (**Supplementary Figure 2**). These findings are in line with other reports, where e.g., the HSP90 inhibitor (NVP) AUY922 shows radiosensitizing effects on GBM spheroid models (40). Also, the HSP90 inhibitor NXD30001, when combined with radiotherapy, significantly inhibited tumor growth and prolonged the median survival in an EGFR-driven genetically engineered mouse model of GBM (41).

In addition, our data demonstrate that combination therapy affects the rate of wound healing in a dose-dependent manner. Interestingly, HSP90 has previously been identified to efficiently decrease migration and invasion of human GBM cell lines by interaction with Ephrin type-A receptor 2 (EPHA2) (42, 43), a protein that was not affected in the performed PEA analysis.

One suggested mechanism for Onalespib’s potentiation on the radiotherapy’s effect could be the disruption of DNA repair. Radiation induces DNA double breaks (DSBs), followed by increased activation of DNA damage repair mechanisms. Counting γH2AX and 53BP1 foci in single cells serves as a sensitive biomarker for DSB presence and the cell’s capacity for DSB repair after exposure to genotoxic agents. H2AX activation and 53BP1 recruitment to DSB sites, facilitated by its Tudor domain, plays a critical role in the DSB repair process by forming repair foci and activating cell cycle checkpoints to provide more time for repair. Quantifying these foci through nucleus immunofluorescence staining and microscopy reveals the extent of DNA damage and repair activity within individual cells.

Our finding demonstrates that Onalespib can increase the amount of DSBs as measured by γH2AX and 53BP1 foci in monotherapy, an effect that could be attributed to the inhibition of proteins involved in various DNA damage response pathways. These pathways include upstream checkpoint signaling, double-strand break repair by homologous recombination (HR), non-homologous end joining, as well as processes such as cross-link repair and DNA replication. Overall, the combination therapy led to lower expression of proteins than in radiotherapy alone, among which several are involved in radiation damage response such as CDKN1A (p21).

The combination treatment of 6 Gy and 100 nM Onalespib resulted in a significant increase in 53BP1 foci in U343 MG but not in U87 MG cells, possibly due to differences in the DNA repair capacity. U87 MG has previously been described as resistant to TMZ treatment due to increased cell cycle arrest and DNA repair response. Notably, Onalespib has been found to effectively deplete key HR proteins, like CHK1 and RAD51, impairing HR repair and making patient-derived glioma stem cell lines more susceptible to radiation and TMZ (15). Studies in zebrafish bearing glioma xenografts have also shown the synergistic effects of Onalespib in combination with the GBM standard treatment, TMZ. Earlier *in vitro* and *in vivo* studies also showed that Onalespib can enhance the TMZ treatment (20). A significant limitation of radiation therapy is its reduced efficacy in hypoxic regions; however, HSP90 inhibition by NXD30001 and NVP-AUY922 has been shown to increase radiosensitivity in hypoxic CD133-positive subpopulations glioblastoma spheroids (10, 40), likely due to HIF-1α inhibition.

Proteomic analysis of U343 MG demonstrated Onalespib’s association with downregulation of proteins involved in several functional pathways, whereas radiation therapy affected both up and down-regulation of these proteins.

Notably, CDKN1A (p21), which was found upregulated due to the combination treatment in our study, suggests interference with pathways critical for tumor suppression and may explain the synergetic effect of Onalespib to radiation. In literature, p21 remains still contradictory with the function either as an oncogene or as a tumor suppressor (44, 45). p21 acts as a regulatory checkpoint in cell division, leading to cell cycle arrest, increased levels of p53, and the activation of DNA repair mechanisms (46). It facilitates this arrest by binding to and inhibiting the activity of CDK1 and CDK2, thereby preventing progression from G1 to S phase and from G2 to mitosis. Downregulation of CDK1 contributes to G2 phase arrest and reduced cell proliferation, consistent with our findings of G2/M phase accumulation in the combination treatment groups. Interestingly, high LET radiation can induce CDKN1A foci at the DSB site that persist for several hours suggesting that CDKN1A can directly mediated interact with proteins involved in DDR (47).

Aggressive tumors are known to produce growth factors that promote the growth of blood vessels (angiogenesis), making endothelial cells proliferate and become more resistant to radiation. VEGFA, a critical factor in promoting angiogenesis, was significantly reduced by both HSP90 inhibition and radiotherapy alone. However, when used together, the combination treatment resulted in an even greater decrease in VEGFA expression, indicating a stronger inhibition of tumor blood vessel formation.

Additionally, TRAIL was slightly lowered by both HSP90 inhibition with Onalespib and radiotherapy independently. Yet, the combination treatment notably increased TRAIL expression. TRAIL plays a crucial role in regulating various biological responses in both cancer and normal cells, including the induction of programmed cell death mechanisms as apoptosis and necroptosis (48). The observed elevated levels of TRAIL in the combination group suggest a heightened activation of cell death pathways and might explain the observed synergistic effects. Previously, HSP90 inhibition by SNX-2112 was reported to enhance TRAIL-induced cytotoxicity on cervical cancer cells (49). This suggests that combining HSP90 inhibition with TRAIL could, besides of combination with radiotherapy, represent a novel treatment strategy, which would involve overcoming apoptosis resistance (49). Current research is directed towards developing anticancer agents that activate TRAIL, as it selectively targets cancer cells with minimal damage to normal cells (50).

The full list of altered protein expression in the Onalespib and combination treated groups (**Supplementary Figure 4**) may also reveal potential therapeutic targets for future investigation. For example, radiotherapy increased the expression of the immune checkpoint molecule CEACAM1. A recent study combining radiotherapy with CEACAM1 inhibitors resulted in strong and enduring immune responses against murine glioma, leading to extended survival in some mice (51). Consequently, targeting CEACAM1 could offer an effective immunotherapy strategy for the treatment of glioma.

The here presented *in vitro* analysis of Onalespib and radiotherapy demonstrated significant reductions in tumor cell growth, migration potential, and disruption of DNA double-strand break (DSB) damage response. These findings highlight the potential efficacy of this combination in treating GBM.

Despite the promising results, this study has several limitations. One significant limitation is the use of the U87 MG cell line obtained from ATCC, which has been shown to differ genetically from the original U87 MG line established at Uppsala University in the 1960s (52). Although the ATCC U87 MG line is likely to be a bona fide human glioblastoma cell line of unknown origin, it is widely used in glioma research due to its well-known characteristics and tumorigenic properties. However, the differences between ATCC U87 MG and the original glioma model suggest caution when comparing findings with studies that do not specify the origin of their U87 MG cells. Future studies should include additional, well-characterized glioma organoid models to strengthen the generalizability and applicability of the results. Additionally, while our OLINK proteomic analysis yielded valuable insights, it remains exploratory. Confirmation of key proteins, particularly those with potential as biomarkers or therapeutic targets, through more traditional methods like Western blotting, is essential for validation. Also, the proteomic investigation represents only a snapshot of the underlying processes and further studies are needed to elucidate the functional implications of these proteomic changes and their potential therapeutic implications for the treatment of GBM. In previous *in vivo* studies conducted by our group, the combination of Onalespib and radiation in models of colorectal, squamous cell carcinoma (12), and neuroendocrine (14) tumors did not result in adverse effects such as behavioral changes, loss of appetite, or weight loss. However, further investigation into Onalespib’s impact on tumor growth and normal brain tissue is essential. Preclinical studies using neural stem or progenitor cells should assess survival, differentiation, and neurogenesis to evaluate potential neurotoxic effects. These studies are crucial to ensure Onalespib’s translational potential for brain cancer treatment, supported by a robust safety profile.

We are encouraged by our promising results, which suggest that the combination of radiation treatment and HSP90 inhibition could be an effective therapeutic option for patients with GBM, particularly those resistant to standard treatments. The synergistic effects of this combination hold promise for improving treatment efficacy and achieving better clinical outcomes. However, further investigations are required to determine optimal dosing and to identify the toxicity profile of Onalespib in a more clinically relevant setting.

## Data Availability

The datasets generated for this study are available on request to the corresponding author.

## Supplementary material

Supplementary Figure 1 Wound healing/migration potential of U343 MG and U87 MG glioblastoma cells. **(A)** 12 h U343 MG LOEWE synergy scores. **(B)** 24 h U343 MG LOEWE synergy scores. **(C)** 12 h U87 MG LOEWE synergy scores. **(D)** 24 h U87 MG LOEWE synergy scores.

Supplementary Figure 2 Live/dead cell percentage and limiting dilution assay. **(A)** U87 MG, U3013MG and U3024MG multicellular spheroids were exposed to Onalespib and radiation and their combination. Data plotted as means ± standard deviation. One-way ANOVA with Tukey’s post-test *(p < 0.05), **(p < 0.01), ***(p < 0.001) and ****(p < 0.0001). **(B)** Limited dilution assay of U87 MG, U3013MG and U3024MG treated with a combination of 25nM, 50nM and 100nM Onalespib and 4 Gy radiation. Spheroid formation efficiency was elevated 3 days after plating. The natural log fraction of non-responding wells was plotted on a linear scale versus the cell density per well.

Supplementary Figure 3 Distribution of γH2AX foci analysis of U343 MG and U87 MG cells. **(A)** Confocal microscopy images of U343 MG cells treated with 100 nM Onalespib and 2 and 6 Gy radiation. Arrows indicate representative instances of counted γH2AX foci. **(B)** Violin plots of U343 MG, number of γH2AX foci per cell. **(C)** Representative images of co-expression 53BP1 and γH2AX foci of U343 MG cells treated with 100 nM Onalespib and 6 Gy radiation. **(D)** Confocal microscopy images of U87 MG cells treated with 100 nM Onalespib and 2 and 6 Gy radiation. Arrows indicate representative instances of counted γH2AX foci. **(D)** Violin plots of U87 MG, number of γH2AX foci per cells. **(F)** Representative images of co-expression 53BP1 and γH2AX foci of U87 MG cells treated with 100 nM Onalespib and 6 Gy radiation. **(G)** Western blot analysis of CDKN1A (p21) and γH2AX after exposure of Onalespib, radiation and their combination.

Supplementary Figure 4 Left: Hierarchical clustering analysis illustrates the most prominent alterations in protein expression levels observed in U343 MG cells treated with radiation, Onalespib, and their combination, relative to untreated control cells. The difference in log(expression) to control (dNDX) is indicated, with positive values highlighted in red, indicating higher expression compared to control, and negative values shown in blue, indicating lower expression. Middle: The standard deviation between treatments, where a large standard deviation indicates differentially expressed proteins of interest. The boxes delineate divergent clusters of interest of proteins with similar expression patterns. Right: Absolute dNDX for each treatment compared to control, using the same scale as left. Black square indicates the combination treatment group, with Onalespib positioned to the left and radiation with 2 Gy on the right-hand side.

Supplementary Figure 5 Uncropped Western blot membranes. Upper row: CDKN1A (p21) and corresponding loading control GAPDH. Lower row: γH2AX and corresponding loading control GAPDH. The dashed line shows the cropped image used in **Supplementary Figure 3**. The box with the solid line indicates a cut of the membrane (for separate incubation with the secondary antibody).
