# Correction: Enhancing glioblastoma therapy: unveiling synergistic anticancer effects of Onalespib - radiotherapy combination therapy

**DOI:** 10.3389/fonc.2026.1823700

**Published:** 2026-03-26

**Authors:** Julia Uffenorde, Mehran Hariri, Eleftherios Papalanis, Annika Staffas, Josefine Berg, Bo Stenerlöw, Hanna Berglund, Christer Malmberg, Diana Spiegelberg

**Affiliations:** 1 Department of Surgical Sciences, Uppsala University, Uppsala, Sweden; 2 Department of Immunology, Genetics and Pathology, Uppsala University, Uppsala, Sweden; 3 Department of Medical Sciences, Uppsala University, Uppsala, Sweden

**Keywords:** CNS tumors, synergy, heat shock protein, radiotherapy, combination therapy, proteomics, proximity extension assay

There was a mistake in **Figure 2D** as published, where the representative images for U87 MG under the control and 10 nM Onalespib conditions were not accurately displayed. The corrected **Figure 2D** appears below.

**Figure 2:**
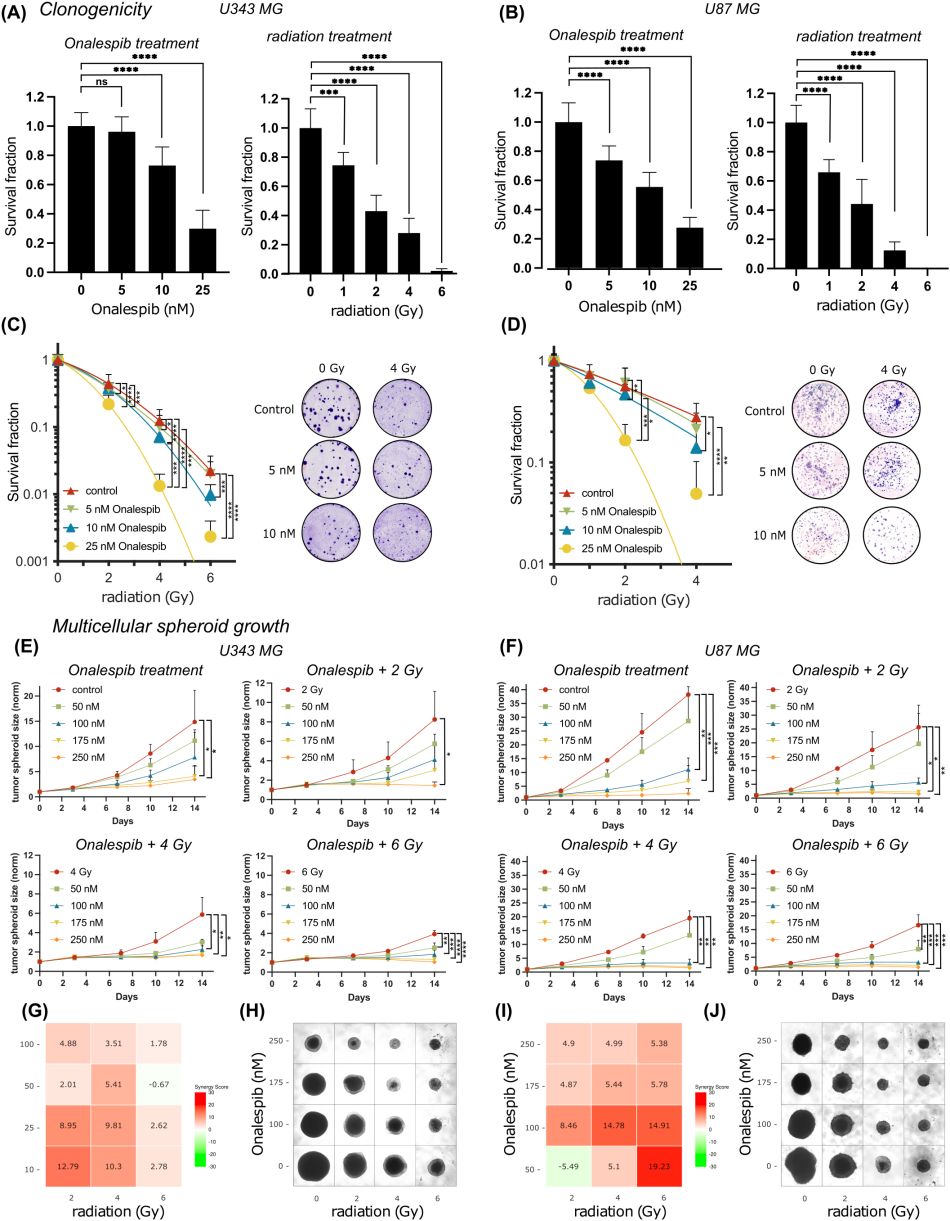
Colony formation and multicellular spheroid growth of U343 MG and U87 MG glioblastoma cells. Survival fraction of Onalespib and radiation treated U343 MG **(A)** and U87 MG **(B)** Survival fraction of Onalespib and radiation combination treatment of U343 MG **(C)** and U87 MG **(D)**. Representative images of the colonies of the monotreatments and the combined treatments of Onalespib and radiation. Onalespib monotherapy and combination therapy with radiation in 3D spheroid model of U343 MG **(E)** and U87 MG **(F)**. Graphs display the normalized spheroid volume (mm3) over time, (means ± standard deviation, n ≥ 3). LOEWE synergy scores for U343 MG **(G)** and U87 MG **(I)**. Representative images of the U343 MG and U87 MG multicellular tumor spheroids at the endpoint of the assay are shown in **(H, J)**, respectively. Data plotted as means ± standard deviation, n ≥ 3. One-way ANOVA with Tukey’s post-test ns (not significant), *(p < 0.05), **(p < 0.01), ***(p < 0.001) and ****(p < 0.0001).

There was a mistake in the caption of **Figure 2** as published. The name of the cell line U343 MG was incorrectly spelled as “U434 MG and U34mg”. The corrected caption of **Figure 2** appears below.

The original version of this article has been updated.

