# Supplementary figures and images for: Enhancing glioblastoma therapy: unveiling synergistic anticancer effects of Onalespib - radiotherapy combination therapy
Source: Front Oncol. 2025 Jan 30;15:1451156. doi: 10.3389/fonc.2025.1451156 (PMC11821960; doi:10.3389/fonc.2025.1451156)

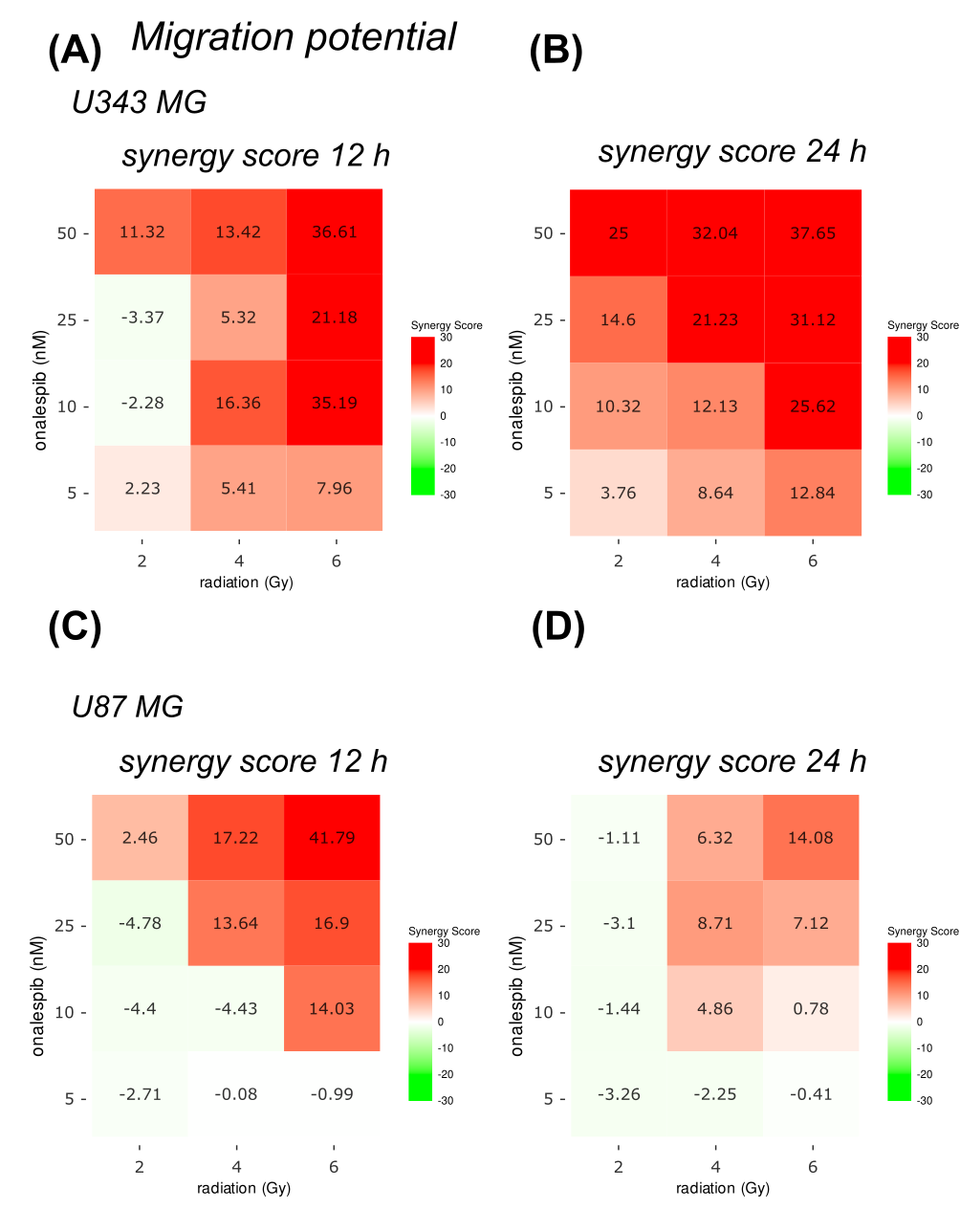

Supplement: Supplementary Figure 1 — Wound healing/migration potential of U343 MG and U87 MG glioblastoma cells. (A) 12 h U343 MG LOEWE synergy scores. (B) 24 h U343 MG LOEWE synergy scores. (C) 12 h U87 MG LOEWE synergy scores. (D) 24 h U87 MG LOEWE synergy scores. [file Image1.png]

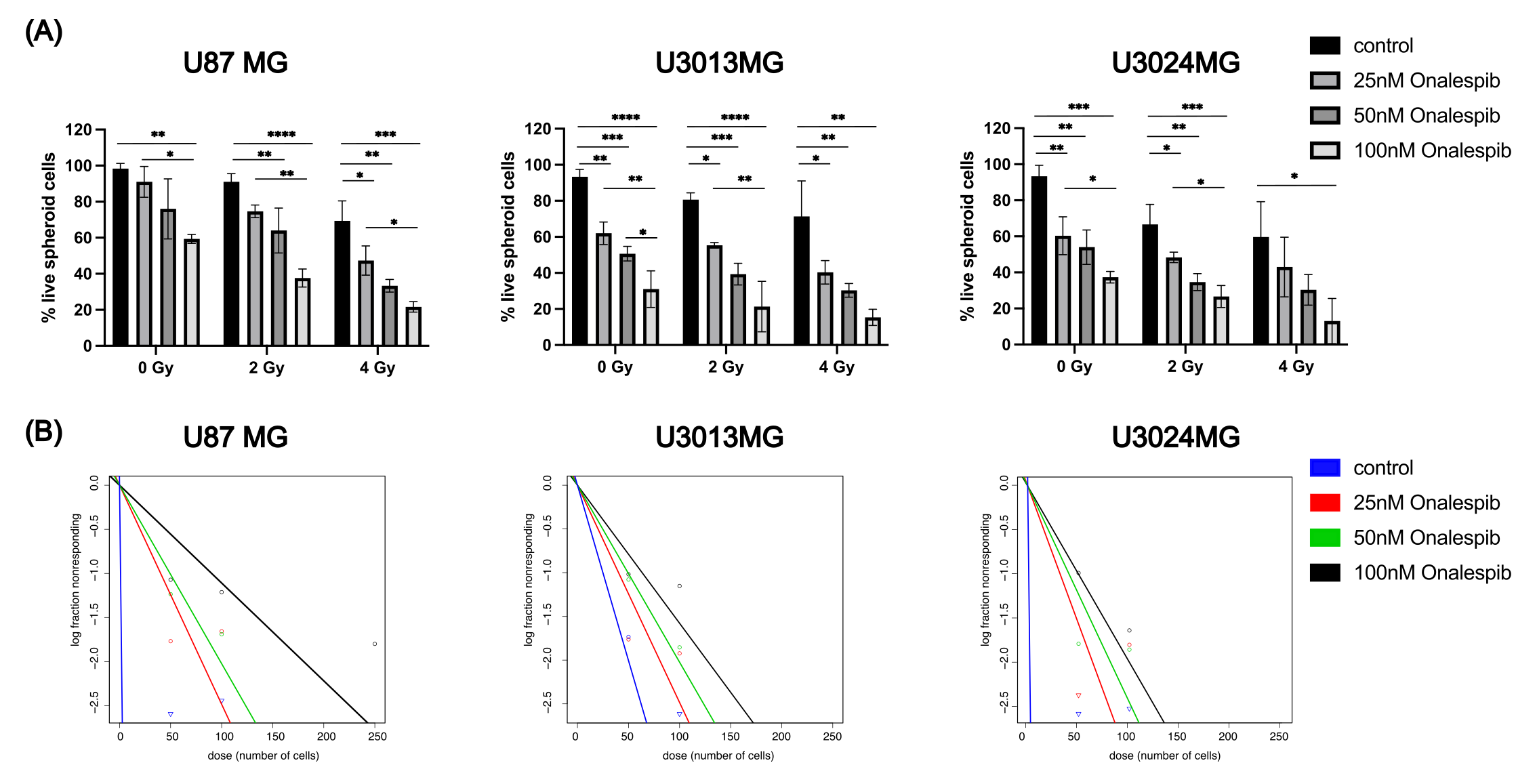

Supplement: Supplementary Figure 2 — Live/dead cell percentage and limiting dilution assay. (A) U87 MG, U3013MG and U3024MG multicellular spheroids were exposed to Onalespib and radiation and their combination. Data plotted as means ± standard deviation. One-way ANOVA with Tukey’s post-test *(p < 0.05), **(p < 0.01), ***(p < 0.001) and ****(p < 0.0001). (B) Limited dilution assay of U87 MG, U3013MG and U3024MG treated with a combination of 25nM, 50nM and 100nM Onalespib and 4 Gy radiation. Spheroid formation efficiency was elevated 3 days after plating. The natural log fraction of non-responding wells was plotted on a linear scale versus the cell density per well. [file Image2.png]

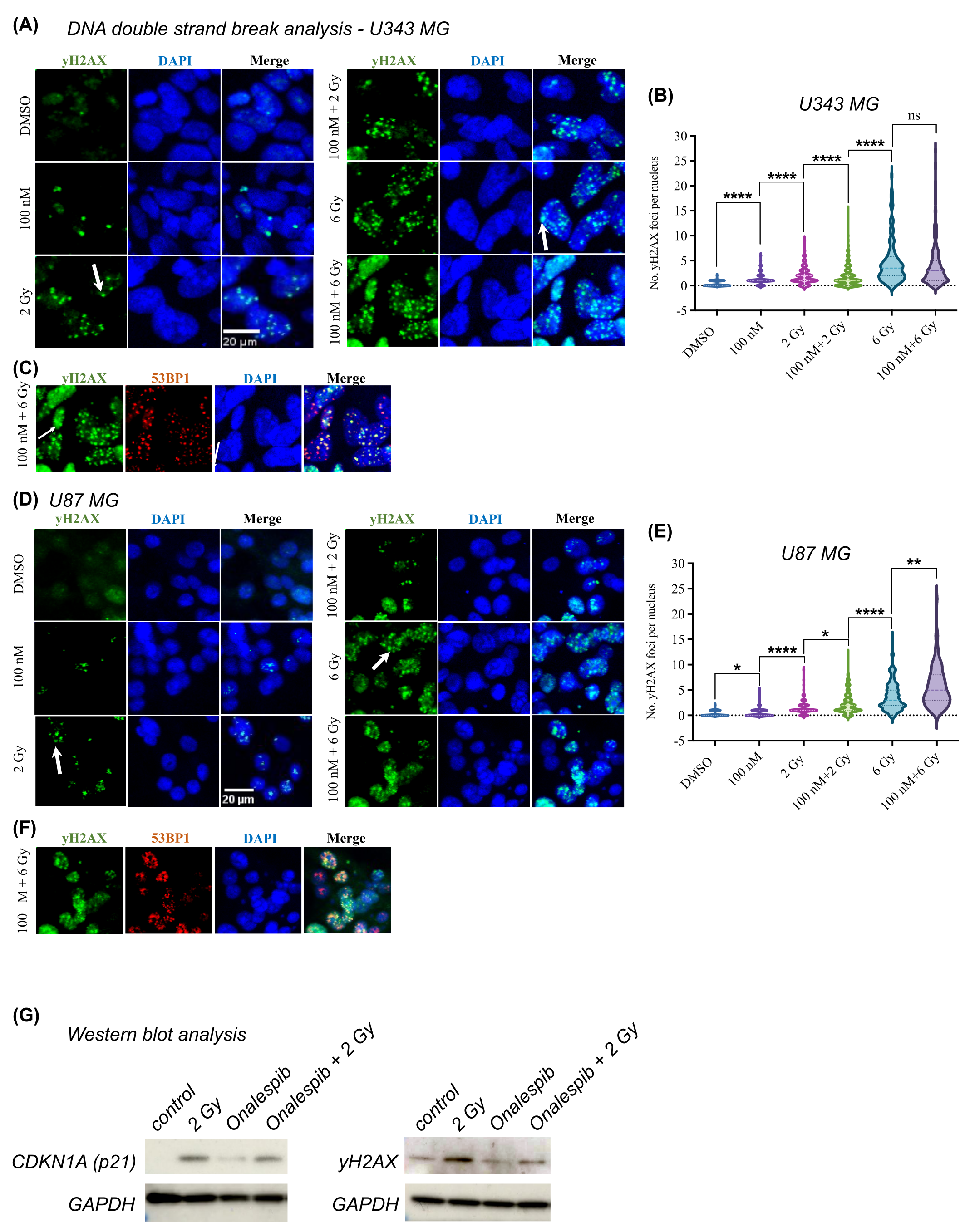

Supplement: Supplementary Figure 3 — Distribution of γH2AX foci analysis of U343 MG and U87 MG cells. (A) Confocal microscopy images of U343 MG cells treated with 100 nM Onalespib and 2 and 6 Gy radiation. Arrows indicate representative instances of counted γH2AX foci. (B) Violin plots of U343 MG, number of γH2AX foci per cell. (C) Representative images of co-expression 53BP1 and γH2AX foci of U343 MG cells treated with 100 nM Onalespib and 6 Gy radiation. (D) Confocal microscopy images of U87 MG cells treated with 100 nM Onalespib and 2 and 6 Gy radiation. Arrows indicate representative instances of counted γH2AX foci. (D) Violin plots of U87 MG, number of γH2AX foci per cells. (F) Representative images of co-expression 53BP1 and γH2AX foci of U87 MG cells treated with 100 nM Onalespib and 6 Gy radiation. (G) Western blot analysis of CDKN1A (p21) and γH2AX after exposure of Onalespib, radiation and their combination. [file Image3.png]

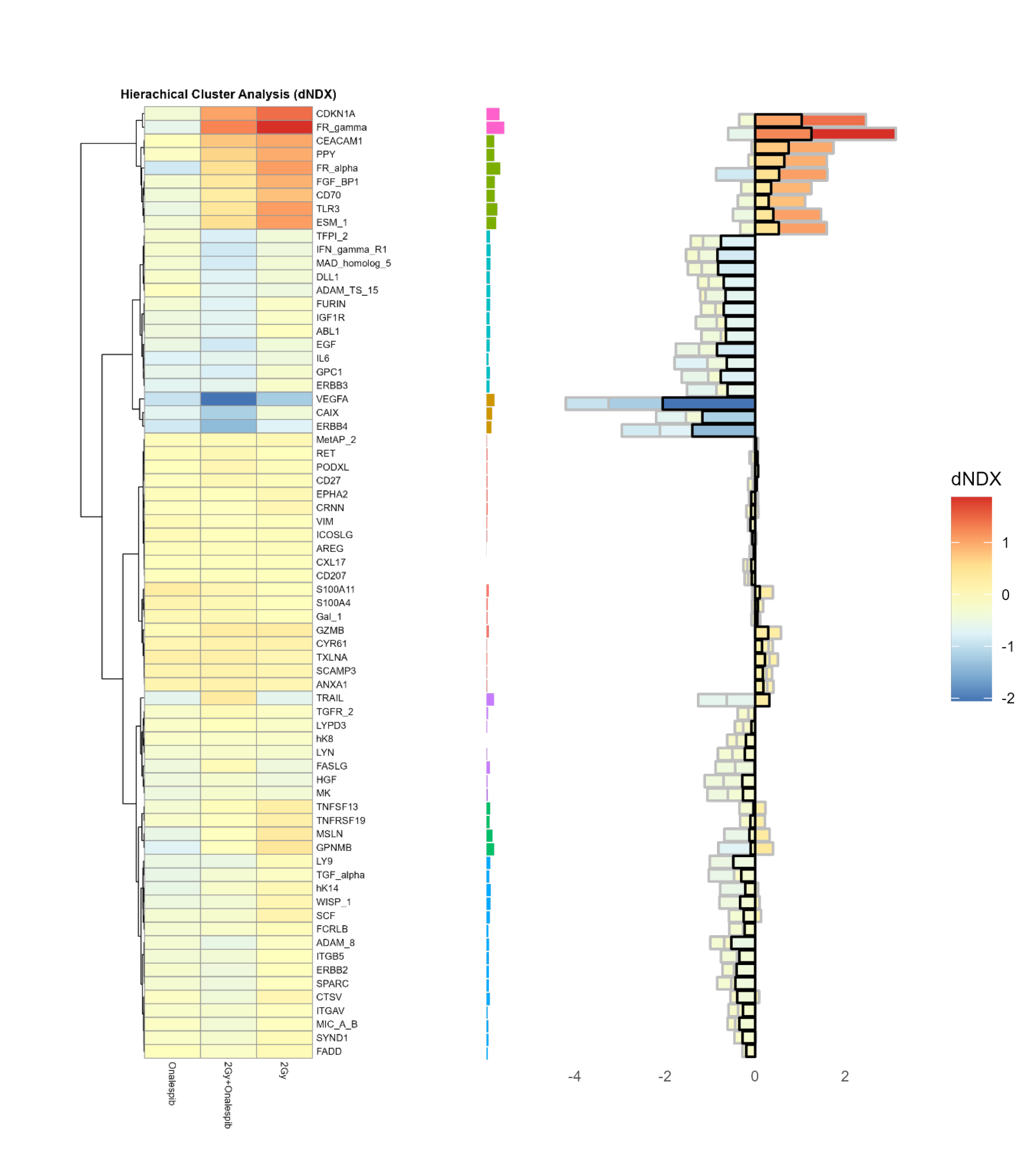

Supplement: Supplementary Figure 4 — Left: Hierarchical clustering analysis illustrates the most prominent alterations in protein expression levels observed in U343 MG cells treated with radiation, Onalespib, and their combination, relative to untreated control cells. The difference in log(expression) to control (dNDX) is indicated, with positive values highlighted in red, indicating higher expression compared to control, and negative values shown in blue, indicating lower expression. Middle: The standard deviation between treatments, where a large standard deviation indicates differentially expressed proteins of interest. The boxes delineate divergent clusters of interest of proteins with similar expression patterns. Right: Absolute dNDX for each treatment compared to control, using the same scale as left. Black square indicates the combination treatment group, with Onalespib positioned to the left and radiation with 2 Gy on the right-hand side. [file Image4.png]

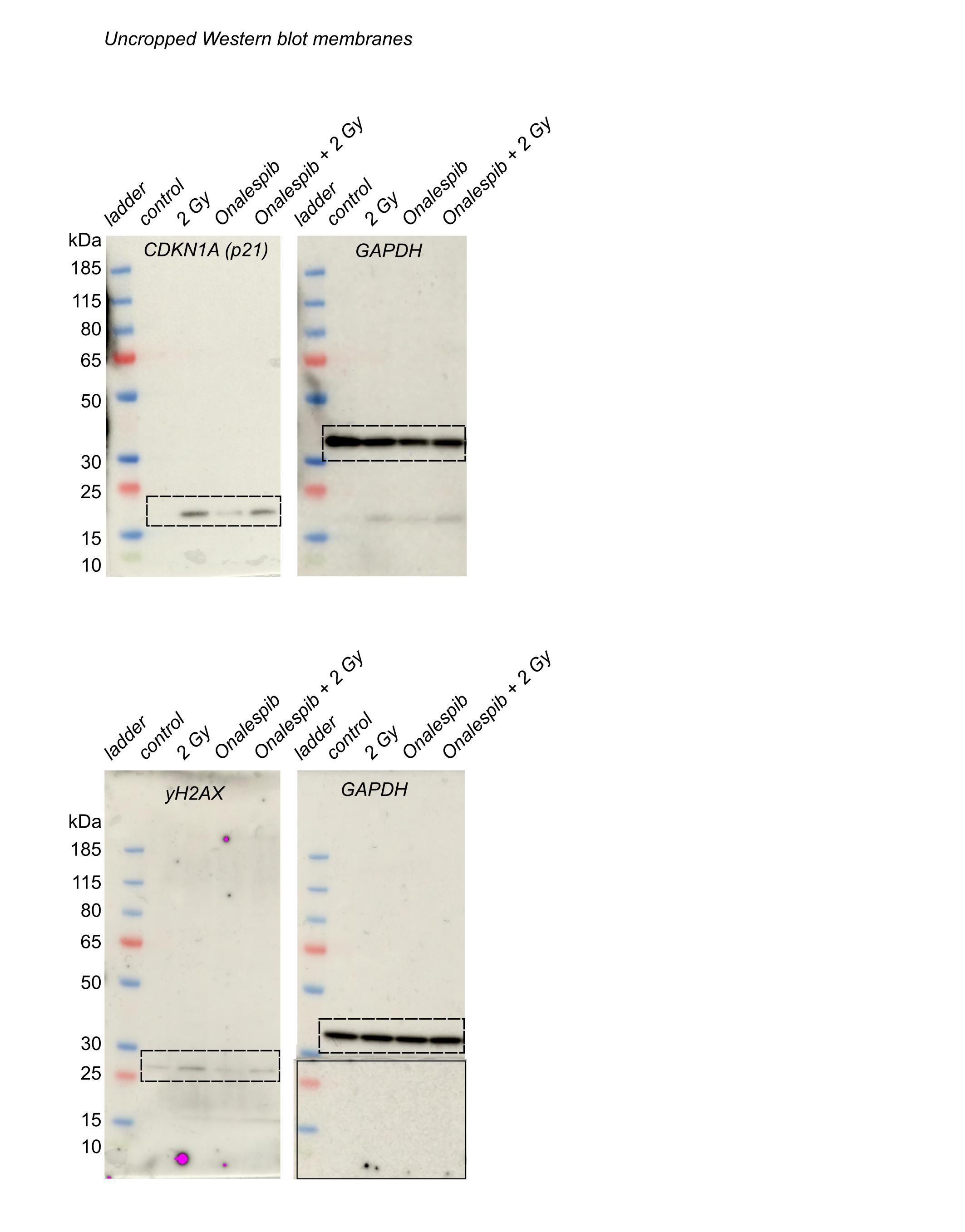

Supplement: Supplementary Figure 5 — Uncropped Western blot membranes. Upper row: CDKN1A (p21) and corresponding loading control GAPDH. Lower row: γH2AX and corresponding loading control GAPDH. The dashed line shows the cropped image used in Supplementary Figure 3. The box with the solid line indicates a cut of the membrane (for separate incubation with the secondary antibody). [file Image5.png]
